# Advancing Clinical and Pathophysiological Insights into Pancreatitis Using Lipidomics and Metabolomics

**DOI:** 10.3390/metabo15100666

**Published:** 2025-10-12

**Authors:** Faizan Ahmed, Xueheng Zhao, Kenneth D. R. Setchell, Maisam Abu-El-Haija

**Affiliations:** 1 Division of Gastroenterology, Hepatology and Nutrition, Cincinnati Children’s Hospital Medical Center, Cincinnati, OH 45229, USA; faizan.ahmed@cchmc.org; 2 Department of Pediatrics, University of Cincinnati College of Medicine, Cincinnati, OH 45221, USA; xueheng.zhao@cchmc.org (X.Z.); kenneth.setchell@cchmc.org (K.D.R.S.); 3 Division of Pathology and Laboratory Medicine, Cincinnati Children’s Hospital Medical Center, Cincinnati, OH 45229, USA

**Keywords:** acute pancreatitis, chronic pancreatitis, metabolites, lipids, metabolomics, lipidomics

## Abstract

Acute pancreatitis (AP) and chronic pancreatitis (CP) are distinct inflammatory conditions with significant clinical burden, including associated complications and mortality. These pancreatic conditions share overlapping pathophysiologic features. Although AP can be followed by recurrent episodes (recurrent acute pancreatitis, RAP), most CP does not follow a simple linear progression from AP; rather, CP reflects sustained processes causing injury to the pancreas (e.g., toxic-metabolic, genetic, obstructive), leading to fibrosis and organ dysfunction. Lipidomics and metabolomics can provide insights into the pathophysiology of the disease. Although researchers have extensively explored lipids and metabolites to better understand disease mechanisms, comprehensive detailed insights into the pathways and intricate roles these molecules play in pancreatitis remain unidentified. This gap can be partially attributed to limited availability of human samples from disease subgroups in pancreatitis, and current technological constraints in analytical methods, particularly regarding complete lipid and metabolite detection, identification, and quantification. In this review, we summarize lipidomic and metabolomic workflows in the context of understanding pancreatitis pathophysiology, including their design and analytical strategies. We also highlight clinical studies on pancreatitis, utilizing lipidomics and metabolomics as a tool to identify altered or dysregulated lipids or metabolites, and their association with the disease state and its progression.

## 1. Introduction

Pancreatitis is an inflammatory condition of the pancreas, representing a clinical condition with significant health effects and potential mortality risk [1]. Acute pancreatitis (AP) and chronic pancreatitis (CP) have occurrence rates of 13–45 and 4–14 per 100,000 individuals, respectively, and they rank among the most frequent diagnoses leading to emergency service visits and hospital admissions in the USA [2,3]. The major etiologies of the condition include gallstones, blockage of pancreatic ducts, smoking, hypertriglyceridemia, abdominal trauma, pharmaceutical agents, infections, genetic mutations, surgical procedures like endoscopic retrograde cholangiopancreatography (ERCP), and alcohol consumption [4,5]. Globally, alcohol is a leading cause of CP and a common cause of AP (second to gallstones in many cohorts), with the notable exception of China, where it ranks third for AP. The initial inflammatory presentation is AP, which is typically reversible. Some patients experience recurrent episodes, termed recurrent acute pancreatitis (RAP) [6]. However, CP does not universally arise from prior AP; instead, CP generally reflects persistent injury to the pancreas that leads to irreversible structural changes, including progressive exocrine and endocrine pancreatic dysfunction, fibrosis, chronic pain, and diabetes mellitus.

Modern ‘omics’ technologies enable comprehensive and high-throughput screening of disease-associated biomarkers in biological samples, with genomics, proteomics, lipidomics, metabolomics, transcriptomics, the microbiome, and combinations thereof emerging as key tools in precision medicine [7]. Genetic variations are inherited from birth, but metabolic profiles are dynamic over a lifetime and can be influenced by other factors such as diet, physical activity, environmental exposure, underlying health conditions, medications, age, gender, and gut microbiota. Metabolomics represents the analysis of a set of low molecular weight molecules (typically < 1500 daltons), while lipidomics encompasses the analysis of biomolecules classified as lipids, which are broadly characterized as biological substances that are typically hydrophobic [8]. Targeted quantification of metabolites enables the accurate examination of metabolic pathways, shedding light on the complex interactions among various biological systems [9].

Advances in metabolomics research on pancreatitis have progressed significantly over recent years, driven by improved sample preparation techniques and analytical methods. However, studies applying metabolomics or lipidomics in pancreatic diseases remain limited, and the literature on metabolic pathways throughout the disease course is lacking but needed. Peng et al. reviewed clinical and murine-based studies that were limited to AP but did not cover cohorts related to CP [10]. Ongoing developments and improvements in analytical techniques and advancements in analytical software necessitate continuous updates to reflect the current status of research on pancreatitis. To address this, we have summarized all current studies on AP and CP using metabolomics and lipidomics. The summary of these studies on pancreatitis aims at increasing our knowledge of key altered metabolites and their pathways, thereby serving as a helpful guide for researchers.

## 2. Workflow of Lipidomics and Metabolomics

There are two primary strategies to study lipids and metabolites that are classified as ‘untargeted’ and ‘targeted’. An untargeted approach is designed to fish out and identify specific systemic lipids or metabolites that differ between different states or conditions when there is no a priori knowledge of what is important. The targeted approach is focused on predefined metabolites/lipids to accurately measure their concentrations [11,12]. Lipids are studied extensively across different domains of medical research, and industrial products [13,14,15]. The widespread use of lipids, including their diverse biological properties, renders them potentially valuable biomarkers in clinical setups and indicators of disrupted pathways under diseased conditions [16,17]. The functional diversity of lipids stems from their structural diversity based on their fatty acid composition, with thousands of potential lipid species possible within a single sample. Due to this structural diversity and complexity amongst different lipid species, it can be challenging to analytically characterize every lipid through lipidomics [18]. Workflows in lipidomics involve sample homogenization, lipid extraction, separation by liquid chromatography, and use of electrospray ionization (ESI, or some other secondary ionization process) high-resolution mass spectrometry (HRMS), detection and identification of lipid features, and statistical analysis [19]. The detailed workflow involves sample collection in the form of biological fluids or tissues, which are then handled and stored after processing. Data acquisition is usually performed using ultra-high performance liquid chromatography coupled with high-resolution mass spectrometry (UHPLC-HRMS) in positive/negative ion mode polarity switching and data-independent collection [20]. Data is processed using feature extraction and multivariate/univariate statistical analysis, which is performed through different available software [21]. Compounds are then identified and annotated using vendor software, online databases, and in-house libraries [22]. Due to the broad physical and chemical properties of lipids, standardized sample preparation protocols are difficult to maintain, and each step of the workflow can significantly influence results. Therefore, tailoring the workflow setups for specific sample types is essential to maintain high quality and reproducible output.

**Figure 1:**
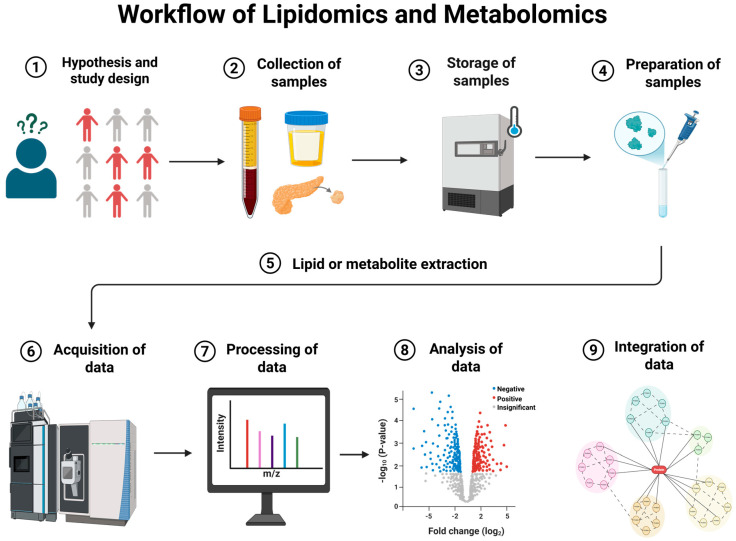
Workflow of lipidomics and metabolomics. (1) Begin by formulating a clear biological hypothesis. A well-structured and optimally designed study is essential to minimize variability and ensure reliable results. (2) Collect samples with precision, ensuring consistency in timing, materials, and reagents to avoid variability. (3) Rapid metabolic reactions necessitate immediate stabilization, such as snap freezing or cooling on ice. (4) Once ready for downstream-processing, process samples appropriately to the analytical platform. (5) Exhaustively extract lipids or metabolites according to analytical platforms and include internal standards and quality control samples. (6) Analyze samples using one or more analytical platforms to generate raw data. (7) Process raw data through multiple steps to accurately identify and annotate compounds. (8) Perform statistical tests to identify significant differences between groups or samples, aligned with the hypothesis and study design. (9) Summarize study findings using pathway and enrichment analysis to further validate specific lipids or metabolites of interest through targeted analysis.

Metabolomics follows a similar workflow, which includes collection of samples, storage, and preparation, followed by acquisition, interpretation, and validation of data [23]. Once the samples are collected and stored following a study design, the samples can be processed for further analysis. The initial step involves data acquisition using techniques like liquid or gas chromatography coupled with MS (LCMS, GCMS, DESI) and nuclear magnetic resonance (NMR) spectroscopy [24,25]. Mass spectrometry is widely used for isolating and characterizing metabolites through their mass to charge (*m*/*z*) ratio and ion intensity data. The analyses are performed using specialized tools and databases like the Human Metabolome Database (HMDB) and the Kyoto Encyclopedia of Genes and Genomes (KEGG), with network visualization aiding interpretation of obtained results [26]. Finally, metabolomics may be integrated with other omics data, offering more detailed insights into disease mechanisms and biological processes. These approaches of integrating multiple datasets can enhance understanding of metabolic changes and link these to genetic, environmental, and other factors, to guide the research towards improving outcomes of a disease [27]. The overall general workflow of lipidomics and metabolomics is illustrated in Figure 1.

## 3. Practical Considerations for Sampling and Timing in AP and CP

Peripheral venous blood is the most used biological source and reflects systemic inflammation; central venous blood (splanchnic) can differ in lipid mediators but is rarely available outside research. Urine is great for non-invasive longitudinal sampling but may capture filtered/renal-handled metabolites and may lack serum dynamics. In AP, pronounced acute-phase shifts (e.g., ketones, free fatty acids, cytokine-driven lipid mediators) occur within hours of symptom onset and often normalize as inflammation resolves. Sampling windows and fasting status should therefore be standardized and reported. In CP, metabolic signatures are subtler and chronic, so assessing stability across visits, meals, enzyme therapy, and glycemic control needs to be considered.

High protease/lipase activity in AP serum can degrade lipids and peptides, so prompt cooling, use of protease/lipase inhibitors where appropriate, and rapid processing (snap-freezing) may mitigate any artifacts. Also, hemolysis and lipemia should be documented and considered during QC. It is also necessary to report anticoagulants (serum vs. EDTA/heparin plasma), storage duration/temperature, and freeze–thaw cycles. Cross study harmonization may benefit from shared internal standards and QC reference materials. Most studies take these factors into account and have reported details extensively in their methodology.

Currently there is little knowledge about specific metabolic biomarkers for AP and CP. To address these limitations, lipidomics and metabolomics emerge as a promising platform, providing comprehensive molecular signatures. These technologies may detect subtle metabolic alterations before abnormal levels of conventional clinical markers are detected, and their multi-parameter nature may allow for stratification of disease severity. Metabolomic profiles can effectively reflect the extent of organ dysfunction and systemic inflammation, while lipid mediators directly involved in the inflammatory cascade can serve as early indicators of disease severity. Furthermore, these approaches enable personalized medicine applications through metabolic profiling of different disease phenotypes and potential prediction of treatment response. Despite significant advances in understanding pancreatitis pathophysiology, critical knowledge gaps remain. Current diagnostic and prognostic tools have limited sensitivity and specificity, particularly in early disease stages. The studies on animal models and humans are largely focused on one type only, and so there is a need to bridge both. The heterogeneous nature of pancreatitis presentations and the complex interplay between genetic, environmental, and metabolic factors are not fully understood. Metabolomics and lipidomics offer unprecedented opportunities to address these gaps by providing comprehensive molecular profiles that can reveal disease mechanisms, identify novel biomarkers, and guide personalized treatment strategies. These omics approaches can elucidate the metabolic consequences of pancreatic dysfunction and inflammation, potentially transforming our approach to pancreatitis diagnosis, prognosis, and therapy.

## 4. Methods

**Figure 2:**
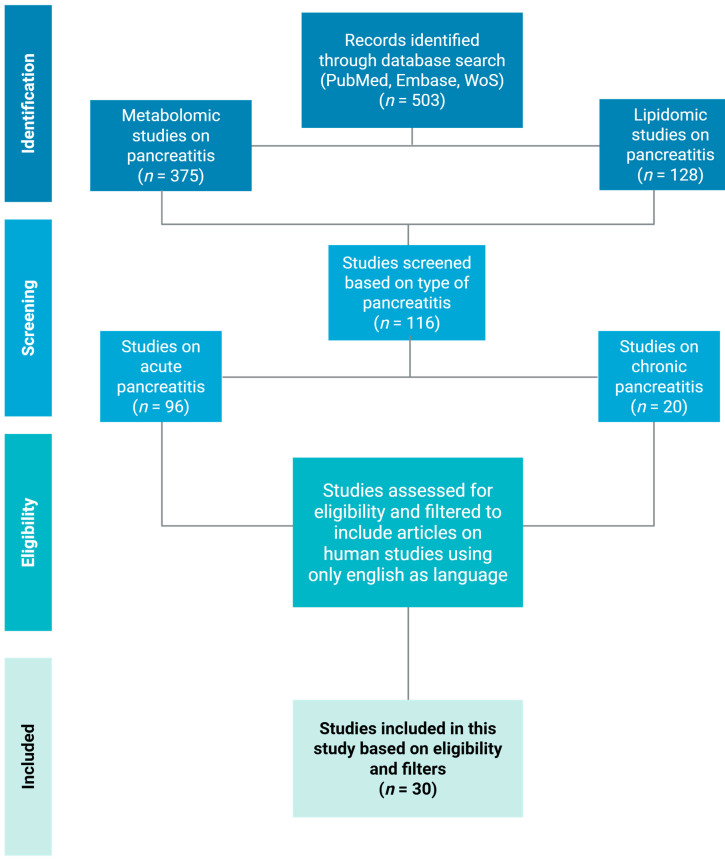
Flowchart showing the search strategy used to filter eligible studies.

This review was based on a PubMed, Embase, and Web of Science literature search for available articles over the years from 2011 to 2025. Two search queries were incorporated. Duplicates were removed. Inclusion criteria were original human studies reporting lipidomics or metabolomics in AP or CP; exclusions included pancreatic cancer, non-English articles, reviews, case reports, and non-human-only studies. After screening the titles, abstracts, and a full text review, 30 human studies relevant to this review were included. The selection process is summarized in Figure 2.

## 5. Lipidomics and Metabolomics Studies on Acute Pancreatitis Patients

### 5.1. Differences in Normal and AP Metabolic States

Untargeted or targeted lipidomics and metabolomics studies on AP have been reported for serum, plasma, and urine samples. Under normal physiological conditions, the pancreas maintains metabolic homeostasis through regulated enzyme secretion and hormonal balance. Metabolomic studies on the serum of AP patients have shown significant disruption in this balance through observed alterations in amino acids, carbohydrates, and lipids. ^1^H-NMR-based metabolomics on AP patients found that acetate, acetone, trimethylamine-N-oxide, and 3-hydroxybutyrate levels were lower during AP, while triglyceride, acetylglycine, isoleucine, and inosine levels were higher in AP patients when compared to healthy controls (HC). This study was primarily focused on identifying novel biomarkers in AP [28]. Hexadecenoic acid levels were observed to be higher in AP patients compared to HC. The results of this study suggested that disruption in fatty acid metabolism plays a role in development of pancreatitis or is a consequence of pancreatitis, potentially leading to both hyperglycemia and hyperglydemia. This connection also explains the increased presence of different sugars, including galactose, mannose, and glucose [29]. Insights into metabolic markers in hyperlipidemic pancreatitis (HLAP) using GC-MS of blood and urine samples revealed HLAP patients had reduced levels of p-hydroxyphenylpropionic acid, p-hydroxyphenylacetate, hippurate, citrate, and aconitate, and elevated levels of fatty acids, tyramine, tyrosine, and tryptophan when compared with HC [30]. Several studies on serum metabolic markers have used advanced analytical techniques and machine learning to facilitate and improve biomarker discovery. GC-MS and random forest plots were used to differentiate between biliary AP (BAP), alcohol-induced AP (AAP), and HLAP from HC, achieving classification accuracies of 0.886, 0.857, and 0.906, respectively, utilizing a five-fold cross-validation. Key metabolites in different types of AP included glycerol-1-hexadecanoate, arachidonic acid, L-tyrosine, D-galactose, L-phenylalanine, erythronic acid, glycine, phosphoric acid, (R)-3-Hydroxybutyric acid, and L-lactic acid, supporting alterations in amino acid and fatty acid metabolism [31]. Similarly to the previous study on BAP and AAP, another study used HPLC-MS to study metabolic signatures in the serum of AP patients and HC. The results showed that metabolites such as pteridine, sterol lipids, fatty acyls, glycerolipids, and glycerophospholipids gave AUROC values above 0.8 [32]. A more recent integrative study on gut microbiota and its association with AP using metabolomic and metagenomic analysis identified around 700 serum metabolites and lipids in each analysis. The study indicated that AP showed 22.9% and 17.7% of the variance in lipidome and metabolome, respectively (*p* < 0.001), and 54.8% of lipids and 41.7% of metabolites were differentially expressed (q < 0.05). The lipid analysis indicated that AP patients had higher triacylglycerols, while HC had a wide range of lipids that were higher [33]. Although serum has been used as the biological fluid for most studies, plasma and urine have also been studied. In order to analyze urinary metabolites, a non-invasive method, proton nuclear magnetic resonance spectroscopy, was used, revealing 60 metabolites. Among these, acetone and ribose were higher in AP compared to the HC [34]. Urinary and plasma metabolites of AP patients were reported to show elevated plasma lipids and choline as well as urine glucose and ketone bodies in these patients. Plasma branched-chain amino acids and urinary hippurate were found to be significantly reduced [35]. In cases of AP, there was a notable decrease in most amino acids, and other compounds such as 3-hydroxybutyric acid, lactic acid, and pyruvic acid. Conversely, carbohydrates such as α-mannopyranoside, glucose, 1,5-anhydro-sorbitol, and lyxose, along with metabolic intermediates like citric acid and oxalic acid, showed increased levels in AP patients [36].

Metabolic profiling of AP against normal states revealed marked disruption in lipid metabolism, with elevated triglycerides, fatty acyls, and specific fatty acids like hexadecenoic and arachidonic acid, while glycerophospholipids displayed varied patterns. Amino acid profiles demonstrate decreased branched-chain amino acids and hippurate, contrasting with increased aromatic amino acids including tyrosine and tryptophan. Carbohydrate metabolism exhibits heightened glucose, mannose, galactose, and other sugars, reflecting pancreatic dysfunction. Organic acid intermediates show bidirectional changes with reduced 3-hydroxybutyrate and elevated lactate and citrate. Ketone bodies present compartment-specific patterns, decreasing in serum while increasing in urine. The findings of this study align with other research on AP, indicating that the primary candidate metabolites for biomarkers are polar molecules, lipids, small organic acids, carbohydrates, and amino acids that play roles in pathways for lipid, glucose, and amino acid metabolism.

### 5.2. Studies Based on Etiology of Acute Pancreatitis

Alcohol-associated AP (AAP) has been accompanied by transient reductions in serum HDL-cholesterol, LDL-cholesterol, and total cholesterol measured during an AP episode, reflecting acute-phase responses with subsequent normalization. In the same cohort, saturated fatty acids, particularly serum palmitic and oleic acids, were increased during hospitalization, whereas α-linolenic acid and several polyunsaturated fatty acids were lower [37]. A study aiming to uncover metabolic responses linked to ERCP-induced AP for prognosis and diagnosis found elevated ketones (acetoacetate and 3-hydroxybutyrate) in serum and urine as a common metabolic response, likely influenced by pre-procedure fasting. Also, differences in serum aspartate and asparagine in post-ERCP subjects were observed, which highlights the effects of interventional procedures on metabolic responses [38].

### 5.3. Studies Based on Severity of Acute Pancreatitis

Different studies have observed altered lipids and metabolites based on the state of severity of disease during an episode of AP. UPLC-HRMS for metabolomic analysis on mild AP (MAP) and cholelithiasis (CHO) patients, including HC, showed 49 significantly altered serum metabolites. Notable findings were elevated levels of glycocholic acid, myristic acid, capryloyl choline, and sphinganine in MAP compared with HC. Similarly, the comparison of CHO against MAP patients showed increased 2-tetradecanone, capryloyl choline, and sphinganine, while L-thyronine and glycocholic levels were reduced in MAP patients [39]. Effects of kynurenine 3-monooxygenase (KMO) inhibition on AP patients showed that activation of the kynurenine pathway (indexed by higher plasma 3-hydroxykyurenine) increased with AP severity. Also, the level of plasma 3-hydroxykynurenine was associated with severity of AP, extent of inflammation, and occurrence of organ dysfunction [40]. When severe AP (SAP) and MAP cases were compared, two key metabolic markers, citric acid and 3-hydroxybutyric acid, were observed as indicators for disease severity classification [29]. SAP poses diagnostic challenges despite advancements in treatment, so extracellular vesicles (EVs) in plasma were quantitatively analyzed using metabolomics from SAP, MAP patients, and HC. A total of 313 metabolites were detected, and four biomarkers, cis-citral, 2-acetylfuran, thiamine triphosphate, and eicosatrienoic acid, showed high diagnostic accuracy (AUC > 0.95), enhancing early SAP severity assessment and detection [41].

It should be noted that there were differences in the metabolic findings based on type of biological fluids, etiology, and severity of AP in these studies. The lipidomics or metabolomics studies showed major differences between AP patients and controls, emphasizing that the metabolic signatures in AP are highly variable and heterogeneous during the disease course. Amino acids, energy-producing organic acids, ketones, sugars, purines, and lipids may be altered during the disease. This may be due to the inflammatory responses observed during the disease, changes in nutritional intakes, or because of differences between individual patients with regard to alcohol intake, diet, and other lifestyle factors. Although these studies reflect different courses or types of AP, the results suggest that major physiological pathways are altered after the onset of disease. It also suggests that during AP, the changes in metabolites or lipids are temporary, and metabolism reverts to normal once the disease symptoms have subsided. The studies may be limited due to the costs involved in performing these assays, as well as difficulties in obtaining samples from large cohorts. These studies also differ with regard to the number of lipids or metabolites identified due to the use of different analytical platforms and identification methods employed, and this highlights a need for consistent analytical methods and identification strategies. A summary of the clinical lipidomic and metabolomic studies on AP patients is shown in Table 1.

## 6. Lipidomics and Metabolomics Studies on Chronic Pancreatitis Patients

CP represents a progressive inflammatory condition characterized by irreversible morphological changes and permanent metabolic dysfunction. Healthy pancreatic function maintains exocrine enzyme production for digestion and endocrine hormone secretion for glucose homeostasis. Normal metabolic profiles include stable amino acid levels, efficient citrate cycle activity, and balanced purine metabolism. During CP, progressive fibrosis and atrophy lead to pancreatic insufficiency, resulting in metabolic alterations. In a metabolomics study designed to generate a non-invasive diagnostic tool for pancreatic cancer which included CP patients, 1H NMR was used to analyze plasma, and significant metabolic alterations in CP patients were observed. CP patients showed increased plasma levels of lipids, formate, creatine, lactate, glucose, and levels of amino acids, i.e., alanine, glutamate, glutamine, histidine, lysine, phenylalanine, and tyrosine. Acetone and 3-hydroxybutyrate were decreased in CP subjects [42]. Two markers were found in a study on urine from CP patients: adenosine was elevated, while citrate levels were decreased in CP compared with controls [34]. More recent studies have focused on analyzing plasma and serum metabolites from subsets of CP patients. A two-phase study focused on identifying and validating a biomarker signature for CP using different MS techniques involved a large cohort of participants. Around 600 serum and plasma metabolites were analyzed, and important metabolites were identified, including beta-carotene, cryptoxanthin, mannose, ceramide (d18:1/24:1), N-acetylcytidine, hippuric acid, indole-3-acetic acid, and behenic acid (C22:0). This research represented the first instance of identifying and independently validating metabolic signatures for diagnosing CP in prospective and extensive cohorts [43]. A 6-metabolite panel of elevated markers were observed in one study, which included phosphatidylserines, phosphatidylcholine, and peptide comprising proline, threonine, arginine, and pentasine, facilitating identification and distinction between the presence and absence of exocrine pancreatic insufficiency (EPI) in affected patients [44].

Several studies have examined neurobiological aspects of pain and effects in CP. One cohort reported a high prevalence of depression that correlated with pain severity; the findings implicated alterations in N-acetyl aspartate and choline in regions linked to emotion and cognition. While CP patients with depression reported worse quality of life (QoL) and distinct plasma metabolomic profiles, causal links between depressive symptoms and specific metabolic signatures remain unclear, and pain during CP may confound these associations [45]. Identification of metabolic serum biomarkers for diagnosing acute CP episodes led to 239 potential biomarkers. Key findings included several specific biomarkers such as diacylglycerol (16:0/18:4), auxin B, carnosic acid, N-(hexacosanoyl)-tetradecasphing-4-enine, and 16-F1-phytoP effectively distinguishing between acute and non-acute episodes of CP, with diacylglycerol (16:0/18:4) demonstrating particularly strong diagnostic performance and showing a high area under the curve of 0.969 in the validation cohort [46].

Untargeted metabolomics was used to compare the serum from Type 3c diabetes mellitus (T3cDM) patients resulting from CP with Type 2 diabetes (T2DM) patients. Significant differences were observed in metabolites such as sphingosine, hippuric acid, bile acids, carnitine, and different types of lipids between T3cDM and T2DM patients. These metabolites were primarily associated with pathways related to sphingolipid metabolism, fatty acid biosynthesis, and bile acid metabolism [47]. A recent study on T3cDM resulting from CP addressed the gap in knowledge on detection of early glycemic changes in the patients using plasma metabolomic profiling. The study utilized LC-MS to create both targeted and untargeted metabolomic profiles in CP patients, and biomarker signatures were derived using logistic regression, CombiROC, and Rapidminer to validate initial findings. LPA (16:0), LPE (16:0), PA (32:0), LPE (22:6), DAG (33:2), PC (37:6), PC (36:5), PC (32:1), PC (36:5), PC (32:1), PE (34:1), PE (38:7), Cer (d18:1/18:0), Cer (d18:1/24:1), and Cer (d18:2/24:1) were higher in CP with T3cDM against controls. Hexanoyl carnitine, deoxycholic acid, Cer (d18:1/16:0), LPE (16:0), LPC (20:3), PE (38:7), PA (32:0), Cer (d18:1/24:1), LPE (22:6), PE (34:2), PE (36:3), PC (37:6), PC (36:5), PC (32:1), PC (36:5), PC (32:1), PE (34:1), and Cer (d18:2/24:1) were significantly higher in non-diabetic CP against controls. Non-diabetic CP patients with glycemic changes (pre-diabetic CP) against non-diabetic CP showed higher LPI (20:4) and PG (34:6). Similarly, CP with T2DM against CP with T3cDM was differentiated by higher SM (d18:1/16:0), DAG (33:4), SM (d18:1/18:1), GlcCer (d18:1/16:1), PS (28:2), PC (29:1), PE (34:2), DAG (33:2), PE (36:3), and PC (34:1). This study helped to create a metabolite panel combined with pancreatic morphology that could detect progression of glycemic changes prior to HbA1c in CP patients [48]. Table 2 provides a summary of available studies in CP.

Compared to AP, research on lipidomics and metabolomics in CP is currently limited due to the complex nature of the disease. CP arises from a combination of genetic, environmental, and other factors, which leads to a diverse patient disease presentation that complicates the identification of distinct metabolic profiles [9]. Additionally, the size of the patient population for CP is often smaller and more heterogeneous, making it challenging to obtain consistent data.

## 7. Altered Metabolic Pathways in Acute and Chronic Pancreatitis

Both inflammatory conditions of AP and CP disrupt normal functioning of the pancreas and influence metabolomic and lipidomic pathways involving carbohydrates, proteins, and lipids. Metabolomic changes that have been identified include variations in amino acids and organic acids, reflecting increased protein breakdown or defective energy metabolism. Similarly, lipidomic alterations involve shifts in lipid profiles, with altered pro- or anti-inflammatory lipids creating defective lipid metabolism pathways. Both AP and CP share fundamental metabolic disruptions that reflect their inflammatory nature at different states. These common pathways may provide insights into disease mechanisms and progression from acute to chronic states. Not all studies have delved into the pathway mechanisms, but there are a few studies that have investigated alterations in these metabolic pathways.

### 7.1. Alterations in Amino Acid and Energy Metabolism

Amino acid metabolism is critical for synthesis of proteins and for supporting immune responses, while energy metabolism is key for producing adenosine triphosphate (ATP) and managing oxidative stress in pancreatitis. Both are critical for tissue repair and maintaining cellular functions. Yang et al. showed the decrease in levels of amino acids in AP patients, with significant alterations in amino acid metabolism, lipid metabolism, and glucose metabolism [36]. This dysregulation negatively influences inflammatory responses and pancreatic function [49]. This study also reported elevated citric acid, a tricarboxylic acid (TCA) cycle intermediate, in AP patients, indicating increased glucose metabolism during acute inflammation.

### 7.2. Altered Lipid Metabolism

Lipids were involved in energy-related metabolic processes during AP, which may explain the reason for altered lipid metabolism during acute episodes of pancreatitis [36]. Xiao et al. reported elevated hexadecenoic acid in AP, suggesting disrupted fatty acid metabolism leading to hyperlipidemia [29]. This affects the glycolysis pathway, causing hyperglycemia and carbohydrate levels with perturbed glucose metabolism [50]. Dancu et al. focused on glycerolipids, sphingolipids, fatty acyls, glycerophospholipids, and other lipids due to their relationship with tissue damage and inflammation, suggesting a pattern of disrupted glycerophospholipid metabolism during AP [32]. Sterol lipid and sphingolipid metabolism is involved in maintaining the integrity of membranes and signaling, and this was altered in most AP studies [51,52,53]. The majority of the studies on CP showed significant alterations in lipid metabolism, implying a role of lipid dysregulation in progression of the disease. Wu et al., in their study on serum metabolic profiles in CP, showed that the most prominent pathways in CP groups compared with HC groups were glycerophospholipid and sphingolipid metabolism [46]. Glycerophospholipid metabolism has been associated with chronic inflammation since several lipid metabolites can alter apoptosis and immune response-associated pathways [54]. Sphingolipids are known to play a complex role in influencing chronic inflammation-related diseases by inhibiting lipid absorption in the intestines, activating inflammatory nuclear receptors, and modifying gut microbiota [55]. Additional metabolic pathways identified in CP included those related to linolenic acid and galactose metabolism, which are relevant to mechanisms underlying pancreatitis, particularly oxidative stress and inflammation [56,57]. Qi et al. showed similar results in analysis of post-CP T3cDM subjects, with differential metabolites from sphingolipid metabolism, linolenic and alpha linolenic acid metabolism, bile acid biosynthesis, fatty acid biosynthesis, and beta oxidation of very long chain fatty acids as the enriched pathways [47].

### 7.3. Etiology-Specific Pathway Alterations

Huang et al. identified distinct metabolic signatures based on pancreatitis etiology [31]. BAP patients showed alterations in nitrogen metabolism, propanoate metabolism, glycolysis or gluconeogenesis, thiamine metabolism, and aminoacyl-tRNA biosynthesis. HLAP patients demonstrated similar changes, with the addition of tyrosine and tryptophan biosynthesis alterations. AAP patients showed altered fatty acid biosynthesis pathways [31]. Decreased glycine in BAP caused abnormal bile acid biosynthesis, potentially damaging pancreatic acinar cells [58]. Lipid transport and phospholipid metabolism pathways were altered due to decreased levels of tyrosine in the HLAP group [59]. 3-hydroxybutyric acid, often linked to dyslipidemia disorders, was reduced in the HLAP and AAP groups, indicating dysregulated fatty acid metabolism [60]. Zhao et al. revealed hypertriglyceridemia’s (HTG) impact on metabolic pathways, showing marked effects on gut microbiota metabolic activity (hippurate, p-hydroxyphenylacetate), tyrosine metabolism, fatty acid metabolism, and the TCA cycle [30]. These gut microbiota alterations correlate with AP severity through dysbiosis, bacterial translocation, and toxic metabolite production [61].

### 7.4. Alterations in Inflammatory, Oxidative Stress and Disease Progression Pathways

Pancreatitis manifests through distinct phenotypes, each characterized by specific metabolic alterations that provide insights into disease mechanisms and progression. The inflammatory responses are accompanied by enhanced oxidative stress pathways, evidenced by increased lipid peroxidation markers and decreased antioxidant metabolites such as glutathione and ascorbate. The interplay between these pathways creates a self-perpetuating cycle of inflammation and oxidative damage that characterizes acute inflammatory responses in pancreatitis [62]. The fibrotic phenotype, particularly prominent in CP, was demonstrated by significant alterations in collagen metabolism and TGF-β signaling pathways [63]. These changes are reflected in modified amino acid profiles related to fibrosis and altered hydroxyproline/proline ratios, indicating active tissue remodeling and fibrosis development [64]. Concurrently with these changes, it further leads to metabolic stress created by disrupted energy metabolism, including impaired glucose utilization and altered TCA cycle intermediates [65]. These metabolic stress pathways are further complicated by abnormalities in lipid metabolism, particularly in fatty acid oxidation and altered phospholipid profiles.

The necrotic phase during pancreatitis presents with distinct metabolic signatures related to cell death processes, including elevated nucleotide breakdown products and increased calcium signaling metabolites. This is often accompanied by enhanced proteolytic activity, reflected in altered trypsinogen activation peptides and modified protein degradation products [66]. In cases where systemic inflammatory responses predominate, significant alterations occur in amino acid metabolism, particularly in branched-chain amino acids and the tryptophan-kynurenine pathway, alongside disruptions in acute phase response markers [67,68]. Endocrine dysfunction, particularly in chronic cases, manifests through impaired glucose homeostasis and disrupted lipid signaling. These changes are evidenced by alterations in the insulin–glucagon axis metabolites and modified incretin pathway markers, accompanied by significant changes in sphingolipid and phospholipid metabolism during pancreatitis [69,70]. These inflammation-based alterations and disease progression pathways during pancreatitis often overlap and interact, creating complex phenotypic presentations that vary based on disease state (acute or chronic), etiology, individual patient factors, and the presentation of other complications.

## 8. Emerging Patterns and Clinical Implications

**Figure 3:**
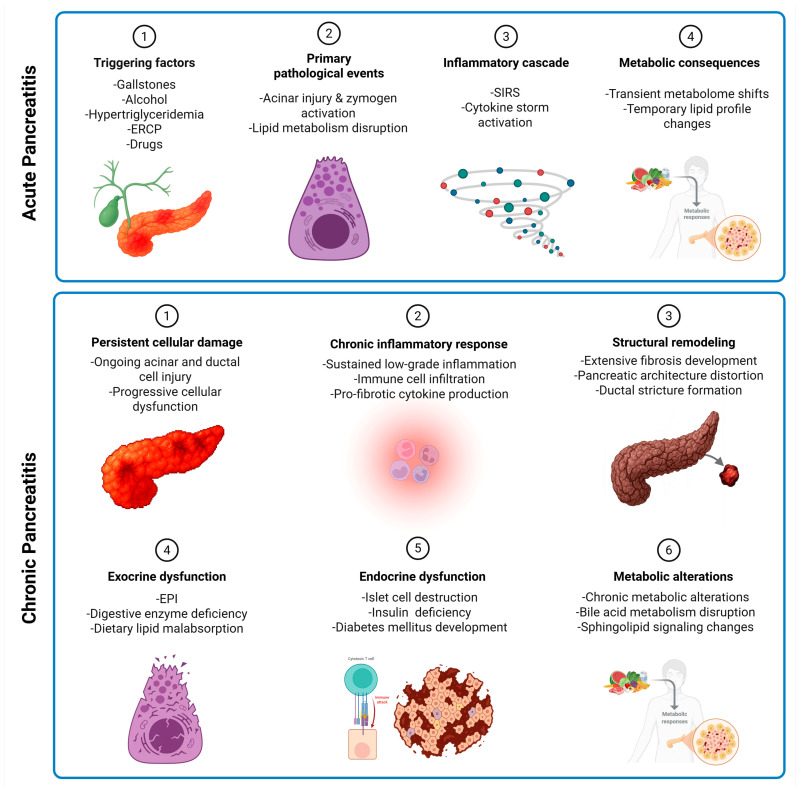
Mechanistic overview of acute and chronic pancreatitis. The figure represents a conceptual mechanism of AP, highlighting adipose lipolysis, systemic inflammation, and transient metabolomic/lipidomic shifts, and CP, highlighting persistent injury, fibrosis, malabsorption, and chronic metabolic alterations. Abbreviations: ERCP: endoscopic retrograde cholangiopancreatography; SIRS: systemic inflammatory response syndrome; EPI: exocrine pancreatic insufficiency.

This review reveals several consistent patterns across studies that provide novel insights into pancreatitis pathophysiology. The mechanisms of metabolic and lipidomic alterations in AP and CP from triggering factors through metabolic changes is highlighted in Figure 3. Specific metabolite panels consistently predict disease severity across multiple cohorts. The combination of LPC 16:0 depletion, 3-hydroxykynurenine elevation, and lactate accumulation predicts severe AP with 85–90% accuracy, outperforming traditional markers like CRP and APACHE II scores. Metabolic changes follow predictable temporal patterns. In AP, lipid alterations occur within 24 h, amino acid changes by 48 h, and energy metabolism disruption peaks at 72 h. This temporal sequence suggests therapeutic windows for targeted interventions. Alcohol-induced pancreatitis shows distinct metabolic signatures compared to gallstone or hypertriglyceridemic causes. Alcohol-related cases exhibit greater oxidative stress markers, more pronounced amino acid disturbances, and persistent metabolic dysfunction, even after clinical recovery. Any transition from AP to ARP to CP involves progressive metabolic reprogramming. Early markers of progression include persistent elevation of branched-chain amino acids, progressive citrate depletion, and accumulation of purine metabolites. These findings enable risk stratification for patients likely to develop CP. Finally, metabolic alterations directly correlate with clinical outcomes. Patients with significant HDL reduction may develop organ failure more frequently. Those with persistent amino acid elevations at discharge may have higher readmission rates, and energy metabolism markers may predict length of hospital stay and ICU requirements.

## 9. Conclusions and Future Directions

The integration of lipidomics and metabolomics into clinical studies on pancreatitis has revealed a large dataset of biochemical alterations and disrupted metabolic pathways that deepen our understanding of the complexity of the disease. In AP, the identification of altered lipid metabolic profiles, such as elevated triglycerides, alongside disrupted amino acid and glucose metabolism, reveals the complicated nature of the disease. CP has been characterized by distinct shifts in lipid and metabolite levels, including beta-carotene and phosphatidylserines that hold promise for diagnostic advancements.

Integrated lipidomics and metabolomics analysis is still in the early stages, especially in the context of pancreatitis research. Priority areas for future research include the development of metabolite-based diagnostic panels for early detection, identification of prognostic markers to predict disease severity, discovery of therapeutic targets through pathway analysis, and personalized treatment strategies based on individual metabolic profiles. The most clinically relevant findings to date include bile acid dysregulation as a potential therapeutic target, sphingolipid alterations as prognostic markers, and amino acid metabolism disruption as indicators of pancreatic dysfunction.

Major challenges include the identification of unknown compounds and accurate detection of an expanded set of metabolites for various metabolic pathways. Nevertheless, the comprehensive analysis of lipidomic and metabolomic profiles in AP and CP reveals an untapped potential for precision medicine. Future study designs should integrate temporal metabolite sampling, standardized analytical platforms, and machine learning approaches to delineate disease-specific molecular signatures. Stratification of patient cohorts based on etiology, coupled with longitudinal metabolic profiling, could unveil distinct biochemical patterns that differentiate disease progression pathways. This systematic approach would not only enhance our understanding of pancreatitis pathophysiology but also facilitate the development of targeted personalized interventions. Ultimately, the translation of metabolic biomarkers into clinical practice requires validation through large-scale, multi-center studies with standardized protocols and robust statistical frameworks. Such efforts will be critical in incorporating metabolomics and lipidomics analysis into clinical settings of pancreatitis care.

## Figures and Tables

**Table 1:** Lipidomic and metabolomic studies on acute pancreatitis (AP) patients.

Reference	Sample Types	Groups and Number	Number of Lipids or Metabolites Identified	Lipids/Metabolites Different in AP vs. Other Groups	Prediction Based on Modeling	Pathway or Enrichment Analysis
Ouyang, 2012 [28]	Serum	AP (*n* = 17), HC (*n* = 23)	NA	(Up): Isoleucine, acetlyglycine, triglyceride, inosine (Down): 3-hydroxybutyrate, trimethylamine-N-oxide, acetate, acetone	NA	NA
Khan et al., 2012 [37]	Serum	At hospitalization: AAP (*n* = 19), HC (*n* = 20)	NA	(Up): Palmitic acid C16:0, monounstaurated fatty acids, oleic acid C18:1n9 (Down): Myristic acid, linoleic acid C18:2, gammalinolenic acid C18:3, homogammalinolenic acid C20:3, alphalinolenic acid C18:3, mead acid C20:3n9	NA	NA
		At 18–24 months: AAP (*n* = 16), HC (*n* = 20)	NA	(Down): Myristic acid, Stearic acid C18:0, homogammalinolenic acid C20:3, alphalinolenic acid C18:3, mead acid C20:3n9	NA	
Lusczek et al., 2013 [34]	Urine	AP (*n* = 5), HC (*n* = 5)	60	(Up): Acetone, Ribose	NA	NA
Villaseñor et al., 2014 [35]	Plasma	AP (*n* = 15), non-AP (*n* = 21)	NA	(Up): Choline, glucose, scyllo-inositol, lipid CH3CH2, lipid (CH2)n, lipid CH2CH═CH, acetone, D-3-hydroxybutyrate, acetoacetic acid (Down): Valine, alanine,	AUC = 0.86	NA
	Urine		NA	(Down): Hippurate, creatine, guanine	AUC = 0.91	
Yang et al., 2016 [36]	Plasma	AP (*n* = 13), HC (*n* = 10)	LC-GCMS: 206; GCMS only: 169	(Up): β-Alanine, inosine, D-sorbitol, D-gluconic acid, L-threitol, D-glucose, D-glucose, arachidonic acid, citric acid, L-glutamine, urea, linolenic acid, myo-inositol, glyceric acid, tetradecanoic acid, cis-9-hexadecenoic acid, L-proline, tyrosine, uric acid, oxalic acid, 2-hydroxypyridine, hexadecanoic acid, glycolic acid, L-tyrosine (Down): L-valine, trans-9-octadecenoic acid, 11-trans-octadecenoic acid, cholesterol, glycylglycine, glycine, dl-isoleucine, L-serine, L-tryptophan, L-isoleucine, L-aspartic acid, phenylalanine, D-fructose, L-proline, L-leucine, D-(−)-lactic acid, L-alanine, L-serine, L-ornithine, 9,12-octadecadienoic acid (Z,Z), L-valine, phosphate, L-leucine, glutamic acid, pyruvic acid	NA	Amino acid metabolism, glucose metabolism, lipid metabolism
Xu et al., 2016 [39]	Serum	MAP (*n* = 38), CHO (*n* = 26), HC (*n* = 36)	432	MAP vs. HC: (Up): sphinganine, capryloyl choline, glycocholic acid, myristic acid, decanoyl choline, dodecanol, 2-tetradecanone, L-thyronine	AUC = 0.865	NA
				MAP vs. CHO: (Up): Sphinganine, capryloyl choline, myristic acid, decanoyl choline, dodecanol, 2-tetradecanone (Down): Glycocholic acid, L-thyronine		
Skouras et al., 2016 [40]	Plasma	AP (*n* = 57), MAP (*n* = 23), Moderate AP (*n* = 23), SAP (*n* = 9)	NA	(Up): SAP vs. others: 3-Hydroxykynurenine (Down): SAP vs. others: Tryptophan	NA	Kynurenine pathway
Lusczek et al., 2016 [38]	Serum	AP-post ERCP (*n* = 9), no AP-post ERCP (*n* = 18)	46	(Up): β-hydroxybutyrate, acetoacetate, glucose	NA	NA
	Urine		72	(Up): β-hydroxybutyrate, acetoacetate		
Xiao et al., 2017 [29]	Serum	AP (*n*, identification = 40, validation = 14), HC (*n* = 37)	44	(Up): 3-hydroxybutyric acid, citric acid, D-mannose, D-glucose, D-galactose, hexadecenoic acid, serotonin (Down): Phosphoric acid. Glycerol, Serotonin	AUC = 0.9907	Galactose metabolism, glycerolipid metabolism, citrate cycle
		SAP (*n* = 6), MAP (*n* = 8)		3-hydroxybutyric acid, citric acid		
Zhao et al., 2017 [30]	Serum	HLAP (*n* = 24), HC (*n* = 39)	20	(Up): Hexadecanoic acid, eicosanoic acid, octadecanoic acid. (Down): Glycine, alanine, citrate, fumaric acid	NA	Tricarboxylic acid cycle (citrate, aconitate), tyrosine metabolism (tyrosine, phenylalanine, tyramine), gut microbiota metabolic activity (p-hydroxyphenylacetate, hippurate)
	Urine			(Up): Proline, leucine, tyramine, phenylalanine, tyrosine, histidine, octadecanoic acid, hexadecanoic acid. (Down): Glycine, citrate, p-hydroxyphenylacetate, hippurate		
Huang et al., 2019 [31]	Serum	BAP (*n* = 27), HC (*n* = 15)	32	(Up): L-lysine (Down): N-acetyl-D-glucosamine, L-lactic acid, L-valine, (R)-3-hydroxybutyric acid, phosphoric acid, glycine, D-galactose, D-glucose, mannitol, L-tyrosine, D-turanose, octadecanoic acid, myo-inositol, oleic acid, cholesterol, glycerol 1-hexadecanoate	AUC= 0.886	Aminoacyl-tRNA biosynthesis, thiamine metabolism, glycolysis or gluconeogenesis, propanoate metabolism, nitrogen metabolism
		AAP (*n* = 20), HC (*n* = 15)		(Down): L-lactic acid, butyric acid, oxalic acid, (R)-3-hydroxybutyric acid, glycine, L-proline, erythronic acid, L-phenylalanine L-serine, L-threonine, L-glutamine, ornithine, L-tyrosine, octadecanoic acid, hexadecenoic acid, L-tryptophan, linoleic acid, oleic acid, arachidonic acid, cholesterol, glycerol 1-hexadecanoate	AUC = 0.857	Aminoacyl-tRNA biosynthesis, nitrogen metabolism, glycine, serine and threonine metabolism, phenylalanine, tyrosine and tryptophan biosynthesis, fatty acid biosynthesis
		HLAP (*n* = 29), HC (*n* = 15)		(Down): N-acetyl-D-glucosamine, L-lactic acid, glycine, D-glucose, mannitol, L-tyrosine, D-turanose, octadecanoic acid, myo-inositol, L-tryptophan, cholesterol, glycerol 1-hexadecanoate	AUC = 0.906	Nitrogen metabolism, aminoacyl-tRNA biosynthesis, thiamine metabolism, phenylalanine, tyrosine and tryptophan biosynthesis, glycolysis or gluconeogenesis
Lou et al., 2022 [41]	Plasma EVs	SAP (*n* = 50), HC (*n* = 50)	313	(Up): Propylparaben, N-acetylglucosamine 1-phosphate, N-oleoyl glycine, lysoPC 17:0, glycoursodeoxycholic acid, L-saccharopine, glycochenodeoxycholic acid, proline betaine, L-valine (Down): 2-(methylthio)ethanol, cyclamicacid, methylstearate, diphenylamine, ginkgoic acid, 15-oxoETE, 2-(methylthio)benzothiazol, 4-hydroxy-L-glutamic acid, hyodeoxycholic acid	Discovery: AUC = 1.00; Validation: AUC = 0.886	NA
		SAP (*n* = 50), MAP (*n* = 50)	46	(Up): Hippuric acid, phenylacetyl-L-glutamine, 2-(dimethylamino)guanosine, estrone (Down): L-carnitine, nonadecylic acid, 2-(methylthio)benzothiazole, hexyl acetate		
Dancu et al., 2023 [32]	Serum	AP (*n* = 34), HC (*n* = 26)	123	(Up): LPC (20:3), all-trans-Retinyl oleate, DG (37:6), LPE (P-16:0/0:0), PE (30:3), Stearyl linolenate, DG (40:9), TG (57:3), 20:1 Cholesterol ester (Down): Dihydrobiopterin, LPA (20:5), LPC (16:1), LPC (18:0/0:0), (S)-3-hydroxystearic acid	AUC > 0.8 for first 19 metabolites	Glycerophospholipid, sterol lipids, fatty acyls, prenol lipids, glycerolipids, sphingolipids
		BAP (*n* = 6), AAP (*n* = 22)		(Up): Myristyl linolenate, LPC (24:1) (Down): MG (0:0/18:0/0:0), (S)-3-hydroxystearic acid, PC (P-18:0/16:0), all-trans-retinyl oleate, and LPC (O-16:0)	AUC > 0.72 for 3 metabolites	
Liu et al., 2024 [33]	Serum	AP (*n* = 45), HC (*n* = 13)	705 lipids, 775 metabolites	(Up): Carbohydrates, hematoporphyrin, organic acids, triacylglycerols (Down): Bile acid, glycerophosphocholine, LPA	NA	Arginine biosynthesis, butanoate metabolism, valine, leucine and isoleucine biosynthesis, histidine metabolism, arginine and proline metabolism, alanine, aspartate and glutamate metabolism, phenylalanine metabolism, sphingolipid metabolism, phenylalanine, tyrosine and tryptophan biosynthesis, synthesis and degradation of ketone bodies

Abbreviations: AP: acute pancreatitis; HC: healthy controls; AAP: alcoholic acute pancreatitis; MAP: mild acute pancreatitis; CHO: cholelithiasis; SAP: severe acute pancreatitis; BAP: biliary acute pancreatitis; HLAP: hyperlipidemic acute pancreatitis; ERCP: endoscopic retrograde cholangiopancreatography; GCMS: Gas chromatography mass spectrometry; LC-GCMS: Liquid chromatography-gas chromatography mass spectrometry; LPC: lysophosphatidylcholine; LPE: lysophosphatidylethanolamine; MG: monoacylglycerol; DG: diacylglycerol; TG: triacylglycerol; LPA: lysophosphatidic acid; AUC: Area under the curve; NA: Not available.

**Table 2:** Lipidomic and metabolomic studies on chronic pancreatitis (CP) patients.

Reference	Sample Types	Groups and Number	Number of Lipids or Metabolites Identified	Lipid/Metabolites Different in CP vs. Other Groups	Prediction Based on Modeling	Pathway or Enrichment Analysis
Zhang et al., 2012 [42]	Plasma	CP (*n* = 20), HC (*n* = 20)	NA	(Up): glucose, lactate, creatine, formate, lipid glyceryls, tyrosine, phenylalanine, lysine, histidine, glutamine, glutamate, alanine (Down): LDL, VLDL, 3-hydroxybutyrate, acetone	NA	NA
Lusczek et al., 2013 [34]	Urine	CP (*n* = 5), HC (*n* = 5)	60	(Up): Adenosine (Down): Citrate		NA
Adam et al., 2021 [43]	Plasma and Serum	Identification (Plasma): CP (*n* = 80), HC (*n* = 80) Validation 1 (Plasma): CP (*n* = 144), HC (*n* = 204) Validation 2 (Serum): CP (*n* = 49), HC (*n* = 56)	Plasma: 620 Serum: 616	(Up): Mannose, Ceramide (d18:1/ C24:1), Behenic acid (C22:0), N-Acetylcytidine (Down): Beta-carotene, Cryptoxanthin, Indole-3-acetic acid, Hippuric acid	Plasma: AUC = 0.85; Serum: AUC = 0.87	NA
Diaz et al., 2021 [44]	Serum	CP (*n* = 53), EPI (*n* = 32), No-EPI (*n* = 21)	1262	(Up): phosphatidylserines (4), phosphatidylcholine (1), Arg-Thr-Pro, pentasine	AUC = 0.79	NA
Sarkar et al., 2022 [45]	Plasma	CP (*n* = 558), HC (*n* = 67)	HC: 22; CP: 70	(Up): benzoic acid (Down): glycine, sarcosine, L-threonine, cholesterol, aminobutanoic acid	NA	NA
Wu et al., 2023 [46]	Serum	Exploratory: CP (*n* = 18), HC (*n* = 21) Identification: CP (*n* = 50), HC (*n* = 17) Validation: CP (*n* = 23), HC (*n* = 10)	239	(Up): Oleandrin, 1-(9Z-heptadecenoyl)-glycero-3-phosphoserine, 5beta-Cyprinolsulfate, 1-(6Z,9Z,12Z,15Z-octadecatetraenoyl)-glycero-3-phosphate, Fludrocortisone, Atracurium, Gibberellin A51, Lagerstroemine,	AUC > 0.7	Sphingolipid metabolism, glycerophospholipid metabolism, linoleic acid metabolism, galactose metabolism
Qi et al., 2023 [47]	Plasma	T3cDM secondary to CP (*n* = 16), HC (*n* = 12)	Positive mode: 2345; Negative mode: 707	(Up): glycocholate, D- -glucose, glycochenodeoxycholic acid, oleamide (Down): deoxycholic acid, Hippurate, caffeine, indole-3-methyl, γ-linoleic acid, paraxanthine, choline, theophylline, DL-stachydrine	AUC = 0.907	Bile acid biosynthesis, beta oxidation of very long chain fatty acids, linolenic acid metabolism, fatty acid biosynthesis, sphingolipid metabolism
Ketavarapu et al., 2024 [48]	Plasma	Identification: CP (*n* = 96), HC (*n* = 7) Validation 1: CP (*n* = 107), HC (*n* = 26) Validation 2: CP (*n* = 43), HC (*n* = 30)	57	(Up): Hexanoyl carnitine, deoxycholic acid, Cer (d18:1/16:0), LPE (16:0), LPC (20:3), PE (38:7), PA (32:0), Cer (d18:1/24:1), LPE (22:6), PE (34:2), PE (36:3), PC(37:6), PC(36:5), PC(32:1), PC(36:5), PC(32:1), PE (34:1), and Cer (d18:2/24:1) (Down): DAG (33:4), PC (O-34:2), PC(36:3), DAG (33:2), PC (34:1), Cer(d18:1/24:0)	AUC for HC and CP = 0.88 for 7 metabolite panel	NA

Abbreviations: CP: chronic pancreatitis; HC: healthy controls; T3cDM: Type 3c diabetes mellitus; LDL: low-density lipoprotein; VLDL: very low-density lipoprotein; LPC: lysophosphatidylcholine; LPE: lysophosphatidylethanolamine; PE: phosphatidylethanolamine; Cer: ceramides; DG: diacylglycerols; AUC: Area under the curve; NA: Not available.

## Data Availability

No new data were created or analyzed in this study. Data sharing is not applicable to this article.

## References

[1] Lee P.J., Papachristou G.I. (2019). New insights into acute pancreatitis. Nat. Rev. Gastroenterol. Hepatol..

[2] Peery A.F., Crockett S.D., Barritt A.S., Dellon E.S., Eluri S., Gangarosa L.M., Jensen E.T., Lund J.L., Pasricha S., Runge T. (2015). Burden of Gastrointestinal, Liver, and Pancreatic Diseases in the United States. Gastroenterology.

[3] Yadav D., Timmons L., Benson J.T., Dierkhising R.A. (2011). Incidence, prevalence, and survival of chronic pancreatitis: A population-based study. Am. J. Gastroenterol..

[4] Weiss F.U., Laemmerhirt F., Lerch M.M. (2019). Etiology and Risk Factors of Acute and Chronic Pancreatitis. Visc. Med..

[5] Gukovskaya A.S., Pandol S.J., Gukovsky I. (2016). New insights into the pathways initiating and driving pancreatitis. Curr. Opin. Gastroenterol..

[6] Testoni P.A. (2014). Acute recurrent pancreatitis: Etiopathogenesis, diagnosis and treatment. World J. Gastroenterol..

[7] Karczewski K.J., Snyder M.P., Karczewski K.J., Snyder M.P. (2018). Integrative omics for health and disease. Nat. Rev. Genet..

[8] Fahy E., Subramaniam S., Brown H.A., Glass C.K., Merrill A.H., Murphy R.C., Raetz C.R., Russell D.W., Seyama Y., Shaw W. (2005). A comprehensive classification system for lipids. J. Lipid Res..

[9] Mayerle J., Hoffmeister A., Werner J., Witt H., Lerch M.M., Mössner J. (2013). Chronic pancreatitis--definition, etiology, investigation and treatment. Dtsch. Arztebl. Int..

[10] Peng Y., Hong J., Raftery D., Qing X., Dan D. (2021). Metabolomic-based clinical studies and murine models for acute pancreatitis disease: A review. Biochim. Et Biophys. Acta (BBA)—Mol. Basis Dis..

[11] Vinayavekhin N., Saghatelian A. (2010). Untargeted Metabolomics. Curr. Protoc. Mol. Biol..

[12] Roberts L.D., Souza A.L., Gerszten R.E., Clish C.B. (2012). Targeted Metabolomics. Curr. Protoc. Mol. Biol..

[13] Segatto M., Pallottini V. (2020). Facts about Fats: New Insights into the Role of Lipids in Metabolism, Disease and Therapy. Int. J. Mol. Sci..

[14] Hong C.R., Jeon B.J., Park K.-M., Lee E.H., Hong S.-C., Choi S.J. (2023). An Overview of Structured Lipid in Food Science: Synthesis Methods, Applications, and Future Prospects. J. Chem..

[15] Cerone M., Smith T.K. (2021). A Brief Journey into the History of and Future Sources and Uses of Fatty Acids. Front. Nutr..

[16] Summons R.E., Welander P.V., Gold D.A., Summons R.E., Welander P.V., Gold D.A. (2021). Lipid biomarkers: Molecular tools for illuminating the history of microbial life. Nat. Rev. Microbiol..

[17] Wei J., Wong L.C., Boland S. (2023). Lipids as Emerging Biomarkers in Neurodegenerative Diseases. Int. J. Mol. Sci..

[18] Lydic T.A., Goo Y.-H. (2018). Lipidomics unveils the complexity of the lipidome in metabolic diseases. Clin. Transl. Med..

[19] Hyötyläinen T., Orešič M., Hyötyläinen T., Orešič M. (2015). Optimizing the lipidomics workflow for clinical studies—Practical considerations. Anal. Bioanal. Chem..

[20] Liang J., Li J., Zhang J., Rong J., Wang X., Zhao C., Zhang H., Shi H., Wu W. (2023). UHPLC-MS/MS-based untargeted lipidomics analysis of septic patients. Clin. Chim. Acta.

[21] Zhao X., Niu L., Clerici C., Russo R., Byrd M., Setchell K.D.R. (2019). Data analysis of MS-based clinical lipidomics studies with crossover design: A tutorial mini-review of statistical methods. Clin. Mass Spectrom..

[22] Köfeler H.C., Ahrends R., Baker E.S., Ekroos K., Han X., Hoffmann N., Holčapek M., Wenk M.R., Liebisch G. (2021). Recommendations for good practice in MS-based lipidomics. J. Lipid Res..

[23] Yan M., Xu G. (2018). Current and future perspectives of functional metabolomics in disease studies–A review. Anal. Chim. Acta.

[24] Theodoridis G., Gika H.G., Wilson I.D. (2011). Mass spectrometry-based holistic analytical approaches for metabolite profiling in systems biology studies. Mass Spectrom. Rev..

[25] Pan Z., Raftery D., Pan Z., Raftery D. (2006). Comparing and combining NMR spectroscopy and mass spectrometry in metabolomics. Anal. Bioanal. Chem..

[26] Chen Y., Li E.-M., Xu L.-Y., Chen Y., Li E.-M., Xu L.-Y. (2022). Guide to Metabolomics Analysis: A Bioinformatics Workflow. Metabolites.

[27] Chong J., Soufan O., Li C., Caraus I., Li S., Bourque G., Wishart D.S., Xia J. (2018). MetaboAnalyst 4.0: Towards more transparent and integrative metabolomics analysis. Nucleic Acids Res..

[28] Ouyang D. (2012). Metabolomic characterization of human pancreatitis by ¹H-NMR spectroscopy. Hepato-Gastroenterology.

[29] Xiao H., Huang J.-h., Zhang X.-W., Ahmed R., Xie Q.-L., Zhu Y.-M., Cai X., Peng Q.-H., Qin Y.H. (2017). Identification of potential diagnostic biomarkers of acute pancreatitis by serum metabolomic profiles. Pancreatology.

[30] Zhao Y., Jia W., Su M., Qiu Y., Wang X. (2017). Novel biomarkers of hyperlipidemic acute pancreatitis: Metabolomic identification. Asian Biomed..

[31] Huang J.-H., He D., Chen L., Dong C.-Y., Zhang S.-H., Qin Y.-H., Yu R., Ahmed R., Kuang J.-J., Zhang X.-W. (2019). GC-MS based metabolomics strategy to distinguish three types of acute pancreatitis. Pancreatology.

[32] Dancu G., Tarta C., Socaciu C., Bende F., Danila M., Sirli R., Sporea I., Miutescu B., Popescu A. (2023). Unraveling the Metabolic Changes in Acute Pancreatitis: A Metabolomics-Based Approach for Etiological Differentiation and Acute Biomarker Discovery. Biomolecules.

[33] Liu J., Yan Q., Li S., Jiao J., Hao Y., Zhang G., Zhang Q., Luo F., Zhang Y., Lv Q. (2024). Integrative metagenomic and metabolomic analyses reveal the potential of gut microbiota to exacerbate acute pancreatitis. NPJ Biofilms Microbiomes.

[34] Lusczek E., Paulo J., Saltzman J., Kadiyala V., Banks P., Beilman G., Conwell D. (2013). Urinary 1H-NMR metabolomics can distinguish pancreatitis patients from healthy controls. J. Pancreas.

[35] Villaseñor A., Kinross J.M., Li J.V., Penney N., Barton R.H., Nicholson J.K., Darzi A., Barbas C., Holmes E. (2014). 1H NMR Global Metabolic Phenotyping of Acute Pancreatitis in the Emergency Unit. J. Proteome Res..

[36] Yang Q., Sun J., Chen Y.Q. (2016). Multi-dimensional, comprehensive sample extraction combined with LC-GC/MS analysis for complex biological samples: Application in the metabolomics study of acute pancreatitis. RSC Adv..

[37] Khan J., Solakivi T., Seppänen H., Lappalainen-Lehto R., Järvinen S., Ronkainen J., Sand J., Nordback I. (2012). Serum lipid and fatty acid profiles are highly changed in patients with alcohol induced acute pancreatitis. Pancreatology.

[38] Lusczek E.R., Colling K., Muratore S., Conwell D., Freeman M., Beilman G. (2016). Stereotypical Metabolic Response to Endoscopic Retrograde Cholangiopancreatography Show Alterations in Pancreatic Function Regardless of Post-Procedure Pancreatitis. Clin. Transl. Gastroenterol..

[39] Xu H., Zhang L., Kang H., Zhang J., Liu J., Liu S. (2016). Serum Metabonomics of Mild Acute Pancreatitis. J. Clin. Lab. Anal..

[40] Skouras C., Zheng X., Binnie M., Homer N.Z.M., Murray T.B.J., Robertson D., Briody L., Paterson F., Spence H., Derr L. (2016). Increased levels of 3-hydroxykynurenine parallel disease severity in human acute pancreatitis. Sci. Rep..

[41] Lou D., Shi K., Li H.-P., Zhu Q., Hu L., Luo J., Yang R., Liu F. (2022). Quantitative metabolic analysis of plasma extracellular vesicles for the diagnosis of severe acute pancreatitis. J. Nanobiotechnol..

[42] Zhang L., Jin H., Guo X., Yang Z., Zhao L., Tang S., Mo P., Wu K., Nie Y., Pan Y. (2012). Distinguishing pancreatic cancer from chronic pancreatitis and healthy individuals by (1)H nuclear magnetic resonance-based metabonomic profiles. Clin. Biochem..

[43] Adam M.G., Beyer G., Christiansen N., Kamlage B., Pilarsky C., Distler M., Fahlbusch T., Chromik A., Klein F., Bahra M. (2021). Identification and validation of a multivariable prediction model based on blood plasma and serum metabolomics for the distinction of chronic pancreatitis subjects from non-pancreas disease control subjects. Gut.

[44] Díaz C., Jiménez-Luna C., Diéguez-Castillo C., Martín A., Prados J., Martín-Ruíz J.L., Genilloud O., Vicente F., Palacio J.P.d., Caba O. (2021). Untargeted Metabolomics for the Diagnosis of Exocrine Pancreatic Insufficiency in Chronic Pancreatitis. Medicina.

[45] Sarkar S., Sarkar P., Revanth M., Hazarika D., Prasanna A., Pandol S.J., Unnisa M., Jakkampudi A., Bedarkar A.P., Dhagudu N. (2022). Pain, depression, and poor quality of life in chronic pancreatitis: Relationship with altered brain metabolites. Pancreatology.

[46] Wu L., Huang X., Ouyang Q., Liu W., Liu S., Huang Y., Peng Y., Ning D., Tan C. (2023). Serum metabolomics study for acute attack of chronic pancreatitis. Clin. Chim. Acta.

[47] Lin Q., Ye Z., Lin H. (2023). Identification of Differential Metabolites Between Type 2 Diabetes and Postchronic Pancreatitis Diabetes (Type 3c) Based on an Untargeted Metabolomics Approach. Lab. Med..

[48] Ketavarapu V., Addipilli R., Ragi N., Pallera P., Simhadri V., Manne S., Sannapaneni K., Aslam M., Talukadar R., Ch V.D. (2024). Plasma Metabolite Profiling Identifies Nondiabetic Chronic Pancreatitis Patients with Metabolic Alterations Progressing to Prediabetes Before HbA1c. Clin. Transl. Gastroenterol..

[49] Sandstrom P., Trulsson L., Gasslander T., Sundqvist T., von D.U., Svanvik J. (2008). Serum amino acid profile in patients with acute pancreatitis. Amino Acids.

[50] Zuo Y.-Y., Kang Y., Yin W.-H., Wang B., Chen Y. (2012). The association of mean glucose level and glucose variability with intensive care unit mortality in patients with severe acute pancreatitis. J. Crit. Care.

[51] Green C.D., Maceyka M., Cowart L.A., Spiegel S. (2021). Sphingolipids in metabolic disease: The good, the bad, and the unknown. Cell Metab..

[52] Rao R.P., Vaidyanathan N., Rengasamy M., Oommen A.M., Somaiya N., Jagannath M.R. (2013). Sphingolipid Metabolic Pathway: An Overview of Major Roles Played in Human Diseases. J. Lipids.

[53] vanMeer G., Voelker D.R., Feigenson G.W. (2008). Membrane lipids: Where they are and how they behave. Nat. reviews. Mol. Cell Biol..

[54] Zeng C., Wen B., Hou G., Lei L., Mei Z., Jia X., Chen X., Zhu W., Li J., Kuang Y. (2017). Lipidomics profiling reveals the role of glycerophospholipid metabolism in psoriasis. GigaScience.

[55] Norris G.H., Blesso C.N., Norris G.H., Blesso C.N. (2017). Dietary and Endogenous Sphingolipid Metabolism in Chronic Inflammation. Nutrients.

[56] Leung K.S., Galano J.-M., Oger C., Durand T., Lee J.C.-Y. (2021). Enrichment of alpha-linolenic acid in rodent diet reduced oxidative stress and inflammation during myocardial infarction. Free Radic. Biol. Med..

[57] Sheng K., Sheng K., Yang J., Xu Y., Kong X., Wang J., Wang Y. (2022). Alleviation effects of grape seed proanthocyanidin extract on inflammation and oxidative stress in a D-galactose-induced aging mouse model by modulating the gut microbiota. Food Funct..

[58] Woolbright B.L., Dorko K., Antoine D.J., Clarke J.I., Gholami P., Li F., Kumer S.C., Schmitt T.M., Forster J., Fan F. (2015). Bile acid-induced necrosis in primary human hepatocytes and in patients with obstructive cholestasis. Toxicol. Appl. Pharmacol..

[59] Fernstrom J.D., Fernstrom M.H. (2007). Tyrosine, Phenylalanine, and Catecholamine Synthesis and Function in the Brain2. J. Nutr..

[60] Mierziak J., Burgberger M., Wojtasik W., Wojtasik W., Mierziak J., Burgberger M., Wojtasik W. (2021). 3-Hydroxybutyrate as a Metabolite and a Signal Molecule Regulating Processes of Living Organisms. Biomolecules.

[61] Zhou R., Wu Q., Yang Z., Cai Y., Wang D., Wu D. (2024). The Role of the Gut Microbiome in the Development of Acute Pancreatitis. Int. J. Mol. Sci..

[62] Cai Y., Yang F., Huang X. (2024). Oxidative stress and acute pancreatitis (Review). Biomed. Rep..

[63] Shek F.W.-T., Benyon R.C., Walker F.M., McCrudden P.R., Pender S.L.F., Williams E.J., Johnson P.A., Johnson C.D., Bateman A.C., Fine D.R. (2002). Expression of Transforming Growth Factor-β1 by Pancreatic Stellate Cells and Its Implications for Matrix Secretion and Turnover in Chronic Pancreatitis. Am. J. Pathol..

[64] Gabr S.A., Alghadir A.H., Sherif Y.E., Ghfar A.A. (2016). Hydroxyproline as a Biomarker in Liver Disease. Biomarkers in Disease: Methods, Discoveries and Applications.

[65] Sakai A., Nishiumi S., Shiomi Y., Kobayashi T., Izumi Y., Kutsumi H., Hayakumo T., Azuma T., Yoshida M. (2012). Metabolomic analysis to discover candidate therapeutic agents against acute pancreatitis. Arch. Biochem. Biophys..

[66] Teich N., Ockenga J., Hoffmeister A., Manns M., Mössner J., Keim V. (2000). Chronic pancreatitis associated with an activation peptide mutation that facilitates trypsin activation. Gastroenterology.

[67] Mayers J.R., Wu C., Clish C.B., Kraft P., Torrence M.E., Fiske B.P., Yuan C., Bao Y., Townsend M.K., Tworoger S.S. (2014). Elevated circulating branched chain amino acids are an early event in pancreatic adenocarcinoma development. Nat. Med..

[68] Jakkampudi A., Sarkar P., Unnisa M., Patil A., Koutarapu C., Jaggaiahgari S., Naik P., Sarkar S., Prasanna A., Chintaluri S. (2023). Kynurenine pathway alteration in acute pancreatitis and its role as a biomarker of infected necrosis. Pancreatol. Off. J. Int. Assoc. Pancreatol. (IAP).

[69] Shi C., Liu S., Zheng M., Yan F., Xu D., Wang W., Chen J., Shi C., Liu S., Zheng M. (2024). Phospholipid and glycerolipid metabolism as potential diagnostic biomarkers for acute pancreatitis. Lipids Health Dis..

[70] Konończuk T., Łukaszuk B., Żendzian-Piotrowska M., Dąbrowski A., Krzyżak M., Ostrowska L., Kurek K. (2017). Plasma Sphingolipids in Acute Pancreatitis. Int. J. Mol. Sci..

